# Immune and Metabolic Alterations in Liver Fibrosis: A Disruption of Oxygen Homeostasis?

**DOI:** 10.3389/fmolb.2021.802251

**Published:** 2022-02-03

**Authors:** Xinyu Li, Quyan Zhang, Zeyu Wang, Quan Zhuang, Mingyi Zhao

**Affiliations:** ^1^ Department of Pediatrics, The Third Xiangya Hospital, Central South University, Changsha, China; ^2^ Xiangya School of Medicine, Central South University, Changsha, China; ^3^ Transplantation Center, The Third Xiangya Hospital, Central South University, Changsha, China

**Keywords:** liver fibrosis, oxidative stress, hypoxia-inducible factor, immunometabolism, mitochondrial dysfunction

## Abstract

According to the WHO, “cirrhosis of the liver” was the 11th leading cause of death globally in 2019. Many kinds of liver diseases can develop into liver cirrhosis, and liver fibrosis is the main pathological presentation of different aetiologies, including toxic damage, viral infection, and metabolic and genetic diseases. It is characterized by excessive synthesis and decreased decomposition of extracellular matrix (ECM). Hepatocyte cell death, hepatic stellate cell (HSC) activation, and inflammation are crucial incidences of liver fibrosis. The process of fibrosis is also closely related to metabolic and immune disorders, which are usually induced by the destruction of oxygen homeostasis, including mitochondrial dysfunction, oxidative stress, and hypoxia pathway activation. Mitochondria are important organelles in energy generation and metabolism. Hypoxia-inducible factors (HIFs) are key factors activated when hypoxia occurs. Both are considered essential factors of liver fibrosis. In this review, the authors highlight the impact of oxygen imbalance on metabolism and immunity in liver fibrosis as well as potential novel targets for antifibrotic therapies.

## Background

Liver cirrhosis is an irreversible liver fibrosis and was the 11th leading cause of death worldwide from 2000 to 2019 according to the WHO (WHO, 2020). Liver fibrosis is a common pathological pathway for various liver diseases, including viral hepatitis, toxic hepatitis, autoimmune hepatitis, and metabolic and genetic liver diseases (Iredale and Campana, 2017). It is a response to persistent liver injury and is characterized by excessive deposition of extracellular matrix (ECM) (Alegre et al., 2017). In the end stage, most liver fibrosis progresses into liver cirrhosis, with the loss of normal function. Early liver fibrosis is reversible, but once it develops into cirrhosis, it is irreversible and leads to functional failure and many complications, such as portal hypertension. Therefore, the prevention and reversal of fibrosis is the main aim for the treatment of liver diseases (Schuppan et al., 2018).

The death of hepatocytes, activated hepatic stellate cells (HSCs), and inflammation are the main causes of the development of liver fibrosis (Alegre et al., 2017). An imbalance in oxygen supply can lead to injury to organ parenchymal cells to secrete various fibrotic and inflammatory cytokine factors and recruit inflammatory cells to the injured stroma in chronic diseases (Darby and Hewitson, 2016; Liu et al., 2017). Hypoxia is rather common in chronic liver diseases. There are several pathways regulating oxygen homeostasis, and hypoxia-inducible factor-1α (HIF-1α) is one of them. HIF-1α, activated in hypoxia, could regulate the transcription of many genes (Kietzmann, 2019). Increasing evidence shows the role of HIF-1α in the process of liver fibrosis (Strowitzki et al., 2018). On the other hand, it always shows excess oxidative stress, mitochondrial dysfunction, and excessive inflammation in chronic liver diseases. Mitochondria are the center of metabolism. With mitochondrial dysfunction in liver disease, it not only releases mitochondrial danger-associated molecular patterns (DAMPs) but also disrupts oxygen homeostasis. DAMPs are released during the condition of liver injury, activating inflammasomes. These injuries may activate HSCs, myofibroblasts, and other cells to synthesize ECM (Schuppan et al., 2018).

Here, we summarize the molecular mechanism of immune, metabolic, and oxygen homeostasis regulation in liver fibrosis to explore precise therapeutic targets and provide clinical treatment strategies.

## The Molecular Mechanism of Oxygen Homeostasis Regulation

### Mitochondrial Function

Mitochondria are important organelles in the generation of adenosine triphosphate (ATP) from lipid, glucose, and amino acid metabolism. The Krebs cycle in the mitochondrial matrix and oxidative phosphorylation in the mitochondrial inner membrane are important processes in energy production. Mitochondria also play a role in β-oxidation of fatty acids, reactive oxygen species (ROS) generation, cell apoptosis, autophagy, calcium homeostasis, and other biological processes (Boengler et al., 2017).

### HIF Pathway

HIFs are key receptors for detecting cellular oxygen rates, consisting of an oxygen-labile α subunit (HIF1α, HIF2α or HIF3α) and a constitutively expressed β subunit (HIFβ) (Peek, 2020). HIFβ is expressed stably and continuously and does not participate in oxygen detection. HIFα subunits are regulated by oxygen at the posttranslational level. Under normal conditions, HIFα subunits are hydroxylated at specific proline residues by the prolyl hydroxylase domain (PHD) protein (Wong et al., 2013). Then, the von Hippel‐Lindau (VHL)/E3 ubiquitin ligase system recognizes the hydroxylated subunits that induce protein degradation and inactivation (Günter et al., 2017). In hypoxic environments, HIFα subunits are not hydroxylated, which mediates protein stabilization. HIFα subunits heterodimerize with HIFβ subunits to form a nuclear heterodimer, which drives target gene transcription by binding to the hypoxia response element (HRE) (Baik and Jain, 2020).

## The Destruction of Oxygen Homeostasis in Liver Fibrosis

### Role of the Mitochondrial Dysfunction in Liver Fibrosis

#### Mitochondrial Structure and Dynamics and Liver Fibrosis

Mitochondrial dysfunction can be detected in many kinds of liver diseases and is believed to be involved in liver fibrosis (Mansouri et al., 2018). It is characterized by ultrastructural mitochondrial lesions, respiratory chain activity reduction, ATP depletion, excessive ROS levels, and mitochondrial DNA (mtDNA) damage. A recent article reported that mitochondrial dysfunction in peripheral cells along with alterations in metabolites of the urea cycle may act as biomarkers for the progression of fibrosis in nonalcoholic fatty liver disease (NAFLD) (Ajaz et al., 2021). The mitochondrial quality control system is crucial for the maintenance of mitochondria, including mitochondrial biogenesis, fusion and fission, and mitophagy.

Mitochondrial biogenesis is regulated by peroxisome proliferator‐activated receptor‐gamma (PPAR γ) coactivator‐1alpha (PGC‐1α) (Wu et al., 1999), which is a kind of transcriptional coactivator that regulates the expression of several transcription factors, such as nuclear respiratory factor (NRF)-1 and NRF-2 (Bhatti et al., 2017; Kiyama et al., 2018), and mitochondrial transcription factor A (TFAM) (Kang et al., 2007). A recent study proved that astaxanthin attenuated hepatocyte damage and mitochondrial dysfunction in NAFLD by upregulating the FGF21/PGC‐1α pathway (Wu et al., 2020). PGC‐1α, NRF-1, and TFAM were also elevated after melatonin treatment in carbon tetrachloride (CCl4)-treated rats. Melatonin protected against liver fibrosis by upregulation of mitochondrial biogenesis (Kang et al., 2016). Moreover, the antifibrotic effects of pomegranate seed oil (PSO), epoxyeicosatrienoic acid-agonist (EET-A), curcumin, liquiritigenin, and Solanum nigrum (AESN) may be related to the upregulation of PGC‐1α (Zhai et al., 2015; Zhang et al., 2015; Tai et al., 2016; Raffaele et al., 2019; Raffaele et al., 2020).

Mitochondrial fusion is regulated by the fusion proteins mitofusin 1 (Mfn1) and Mfn2 and optic atrophy 1 (OPA1), while mitochondrial fission in mammals is regulated by dynamin-related protein 1 (Drp1) (Ni et al., 2015). The role of PGC‐1α in mitochondrial dynamics has been reported. In oxidative stress-induced mitochondrial damage, the downregulation of PGC‐1α is related to abnormal mitochondrial fission. Hence, PGC-1α overexpression reduced Drp1 protein levels and prevented liver fibrosis (Zhang et al., 2020). Particulate matter ≤2.5 μm (PM2.5) also contributes to mitochondrial dynamics dysfunction by increasing Drp1 and decreasing PGC-1α levels (Wang et al., 2021). The overexpression of HK domain-containing 1 (HKDC1) in the liver induced a defect in mitochondrial function by increasing Drp1 (Pusec et al., 2019). The antifibrotic effect of lipoic acid (LA) may be related to the upregulation of Drp1 (Luo and Shen, 2020).

#### Impaired Mitophagy and Liver Fibrosis

Mitophagy refers to the removal of dysfunctional mitochondria through fusion with lysosomes (Yoo and Jung, 2018). It can be classified into Pink1–Parkin-mediated mitophagy and Parkin-independent mitophagy. Mitophagy mediated by Drp-1 was activated by PM2.5, leading to HSC activation and fibrosis. Chronic CCl4 exposure impaired mitophagy in the liver, and melatonin may attenuate liver fibrosis by elevating PINK1 and Parkin (Kang et al., 2016). Researchers have shown that mitochondrial depolarization (mtDepo) occurs early in mice fed a Western diet (high fat, fructose, and cholesterol) and increases mitophagic burden. Together with suppressed mitochondrial biogenesis and mitochondrial depletion, mitochondrial damage promotes the progression of liver fibrosis (Krishnasamy et al., 2019). It is worth mentioning that Parkin-independent mediators, including Bcl2/adenovirus E1B 19 kDa protein-interacting protein 3 (BNIP3) and NIX, can be regulated by HIF-1, thus removing damaged mitochondria and protecting against ROS accumulation.

#### Impaired mtDNA Homeostasis and Liver Fibrosis

In the case of mitochondrial dysfunction, mitochondrial DAMPs (mtDAMPs) are released to the extracellular space. This can stimulate liver inflammation and promote liver fibrosis. A recent study reported that mtDAMPs released from impaired hepatocyte mitochondria could directly activate HSCs (An et al., 2020). The role of mtDAMPs in liver fibrosis will be discussed in detail later.

#### Reactive Oxygen Species Generation in Mitochondria

The mitochondrial respiratory chain is considered the main source of ROS production. ROS refer to oxygen free radicals and nonradical oxidants (Zorov et al., 2014). It can be produced in both the mitochondrial matrix and the intermembrane space (Bouchez and Devin, 2019). Under normal homeostasis, mitochondria can remove physiological ROS by antioxidant mechanisms and metabolic adaptations (Lee et al., 2019) and thus maintain a balance between ROS production and removal. The antioxidant system includes superoxide dismutase (SOD), catalase (CAT), glutathione (GSH), and other antioxidants (Bhatti et al., 2017).

However, the excessive level of ROS caused by increased ROS production and decreased ROS scavenging may lead to protein, DNA, and lipid damage to mitochondria (Zorov et al., 2014; Sorrentino et al., 2018). Additionally, ROS can activate several pathways. Under hypoxia, with low-intensity ROS, the HIF-1α pathway is activated, leading to metabolic adaptation and subsequently inhibiting ROS production (Finkel, 2012). With moderate intensity of ROS in hypoxia, it can regulate inflammatory reactions, such as NACHT, LRR, and PYD domain-containing protein 3 (NLRP3) inflammasome, and mitogen-activated protein kinase (MAPK) signalling (Win et al., 2018). With a high intensity of ROS, apoptosis and autophagy may occur, preventing more damage.

Considering that changes in mitochondrial physiological processes participate in the development and progression of liver diseases, mitochondria are believed to be a promising treatment target for liver fibrosis.

### Role of the HIF Pathway in Liver Fibrosis

Hypoxia and HIFs have been acknowledged as important drivers of liver fibrosis (Strowitzki et al., 2018). In fact, hypoxia is involved in a variety of liver diseases. Hypoxia levels are elevated in liver cancer and may be involved in destroying the natural immune response and creating an immunosuppressive microenvironment (Yuen and Wong, 2020). In NAFLD, hypoxia may mediate hepatic steatosis and abnormal lipid metabolism (Mesarwi et al., 2019). Hypoxia-mediated abnormal immune and metabolic microenvironments are also key factors in the development of fibrosis and liver cirrhosis.

The interaction of HIF-1α and Rho-associated coiled-coil-forming kinase 1 (ROCK1) promotes cell proliferation and collagen synthesis in rat HSCs under hypoxia (Hu et al., 2018). A recent study also showed that activated HIF-1α or HIF-2α in hepatocytes stimulates upregulation of chemokine ligand 12 (Cxcl12) by converting latent transforming growth factor β (TGF-β) to active TGF-β (Strickland et al., 2020). Cxcl12 is involved in the process of liver fibrosis through chemotaxis and activation of HSCs (Li et al., 2020). These findings indicate that HIF acts as an important regulator of liver fibrosis-targeting HSCs (Tirosh, 2018).

During liver fibrosis, hepatic sinusoidal blood flow disorder and hypoxia lead to the secretion of angiogenic factors by liver intrinsic cells. Pathological angiogenesis destroys the hepatic architecture and aggravates liver fibrosis (Poisson et al., 2017). Recent studies identified that hedgehog signalling promoted prospero homeobox protein 1 (PROX1) expression in liver fibrosis, which inhibited HIF‐1α ubiquitination *via* a deubiquitinase called ubiquitin specific peptidase 19 (USP19). This hedgehog signalling-mediated hypoxia response participates in liver sinusoidal endothelial cell (LSEC) angiogenesis and the activation of HSCs (Feng et al., 2019; Yang et al., 2020). It has also been suggested that activated peroxisomal proliferator receptor γ (PPARγ) in HSCs could inhibit the expression of HIF-1α *via* an SMRT-dependent mechanism. The activation of PPARγ improved angiogenesis and vascular remodelling in carbon tetrachloride (CCl4)-induced liver fibrosis in rats. A possible negative feedback loop was raised in which HIF-1α might induce PPARγ expression in response to hypoxia or pathological stress, and then overexpressed PPARγ would inhibit HIF-1α transcription to limit amplification (Zhang et al., 2018).

## The Oxygen Imbalance Mediated-Immune and Metabolic Alterations in Liver Fibrosis

### Mitochondrial Dysfunction Mediated-Immune Regulation in Liver Fibrosis

Sterile inflammation (SI) is a common response to stress and injury in liver disease (Chen et al., 2018). This is a major process in the development of fibrosis and carcinogenesis (Kubes and Mehal, 2012). In SI, DAMPs, which are usually in the intracellular space, are released to the local microenvironment when infections, metabolic disorders, and other stimuli result in hepatocyte cell death, leading to a wide range of immune responses (Iredale and Campana, 2017). Several DAMPs have been identified, including mtDNA and nuclear DNA, high mobility group box-1 (HMGB-1), ATP and other molecules. HMGB-1 is released mostly by hepatocytes and Kupffer cells (KCs). Recent research confirmed the role of HMGB1 in liver fibrosis. It has been reported that HMGB1 could increase collagen type I production in HSCs *via* the receptor for advanced glycation end-products (RAGE), leading to liver fibrosis. The underlying mechanism was the pMEK1/2/pERK1/2/pcJun signalling pathway (Ge et al., 2018). Furthermore, HMGB1 also links hepatocyte injury to hepatocellular carcinoma (HCC) (Hernandez et al., 2018).

MtDAMPs, including mtDNA and formyl peptides, are released to the extracellular space in the case of ROS-driven mitochondrial membrane permeability transition (Mihm, 2018). They recognize pattern recognition receptors (PRRs) on target cells to modulate the function of antigen-presenting cells (APCs), eosinophils, mast cells, and neutrophils (Krysko et al., 2011). For example, mtDNA recognizes Toll-like receptor 9 (TLR9) and NLRP3, and N-formyl peptides (NFPs) recognize TLR9 (Kubes and Mehal, 2012). Therefore, mitochondria are key stimuli of the innate immune response in liver diseases.

MtDNA is the major part of mtDAMP and is released into the cytosol when mitochondrial dysfunction and apoptosis occur. In different kinds of liver injury, the effects of mtDAMP on immune response is different. In patients with nonalcoholic steatohepatitis (NASH), mtDNA levels have been reported to increase (Garcia-Martinez et al., 2016). Furthermore, it increased in patients with active NASH (NAS 4–8 versus NAS 0–3, *p* = 0.0334) (An et al., 2020). It can activate several pathways (Zhang et al., 2019). The first is NLRP3 inflammasomes. NLRP3 can directly activate HSCs, triggering liver fibrosis (Inzaugarat et al., 2019). Furthermore, it has been reported that in the mouse NASH model, mtDNA released by KCs bound and activated the NLRP3 inflammasome to induce interleukin (IL)-1β and IL-18, triggering proinflammatory responses and resulting in liver fibrosis (Shimada et al., 2012; Pan et al., 2018; Hu et al., 2019). Likewise, KCs induced by palmitic acid (PA) induced mtDNA and activated the NLRP3 inflammasome (Pan et al., 2018). The second is TLR9. In a mouse model of NASH, the plasma level of mtDNA increased, and it could activate TLR9, leading to a proinflammatory response (Garcia-Martinez et al., 2016). Therefore, the activation of TLR9 by mtDNA is believed to play a role in the transition from steatosis to steatohepatitis (Garcia-Martinez et al., 2016). The use of the TLR7/9 antagonist IRS954 could block the ability of mtDNA, resulting in a decrease in steatosis, ballooning and inflammation, serum transaminases, and inflammatory cytokine transcript levels (Garcia-Martinez et al., 2016). This revealed the potential of TLR9 ligand therapies. In acute liver injury induced by acetaminophen (APAP), mtDNA, which binds to TLR9, can induce neutrophil activation and liver inflammation. The crucial mediator is microRNA-223 (miR-223), which acts as a negative feedback loop to limit neutrophil overactivation and liver injury (He et al., 2017). DNA-sensing pathways could induce type I interferon (IFN I) production in liver NPCs, which is related to hepatocyte necrosis (Araujo et al., 2018). The third is cyclic GMP-AMP synthase (cGAS)-stimulator of interferon genes (STING). In a mouse model of NASH, STING deficiency attenuated steatosis, fibrosis, and inflammation in the liver; exposure to a STING agonist led to hepatic steatosis and inflammation in WT mice but not in STING-deficient mice (Yu et al., 2018). STING functions as a mtDNA sensor in KCs and increases the expression of TNF-α and IL-6 through the nuclear factor-κB (NF-κB) signalling pathway in NASH (Yu et al., 2018). The activation of STING in macrophages is also related to liver fibrosis (Luo et al., 2018). Furthermore, other mediators related to mitochondria are formyl peptides. Formyl peptides are released from dysfunctional mitochondria and bind to formyl peptide receptors (FPRs) (Krysko et al., 2011). FPRs are expressed at high levels on neutrophil granulocytes and mononuclear phagocytes (Mihm, 2018). This binding leads to the migration of immune cells to the injured tissue, activating proinflammatory responses (Raoof et al., 2010). The role of formyl peptides has been revealed in systemic inflammatory response syndrome (SIRS) and ischaemia/reperfusion injury (IRI) (Zhang et al., 2010; Hu et al., 2015). However, the direct effect of formyl peptides on liver fibrosis has not been identified.

Moreover, mitochondria are the site of ATP production, and when in disease, high concentrations of ATP may be released extracellularly. ATP binds to P2X7 receptors on neutrophils, inducing NLRP3-ASC-caspase-1 inflammasome and IL-1β secretion (Schroder and Tschopp, 2010; Karmakar et al., 2016).

These studies revealed the crucial role of mtDAMPs in modulating the immune response and liver fibrosis, which are promising biomarkers and treatment targets.

### HIF Mediated-Metabolic Regulation in Liver Fibrosis

An increasing number of studies have confirmed that HIF-1 acts as a crucial regulator in metabolic reprogramming in liver fibrosis (Corcoran and O’Neill, 2016). Glucose transporters 1 (GLUT1) and pyruvate kinase isozymes M2 (PKM2) are confirmed to be the target genes of HIF-1 and the key molecules of the Warburg effect (Wan et al., 2019). Wan et al. (2019) reported that HIF-1 upregulated GLUT1 and PKM2 expression in fibrotic liver and exosomes derived from activated HSCs. Interestingly, exosomes from activated HSCs were absorbed by KCs, LSECs, and quiescent HSCs, which enhanced glycolysis of these liver nonparenchymal cells. These findings represent a novel mechanism: upon injury of parenchymal hepatic cells, HIF-1 can regulate nonparenchymal cells (NPCs) to maintain synchronization of metabolic reprogramming.

**FIGURE 1:**
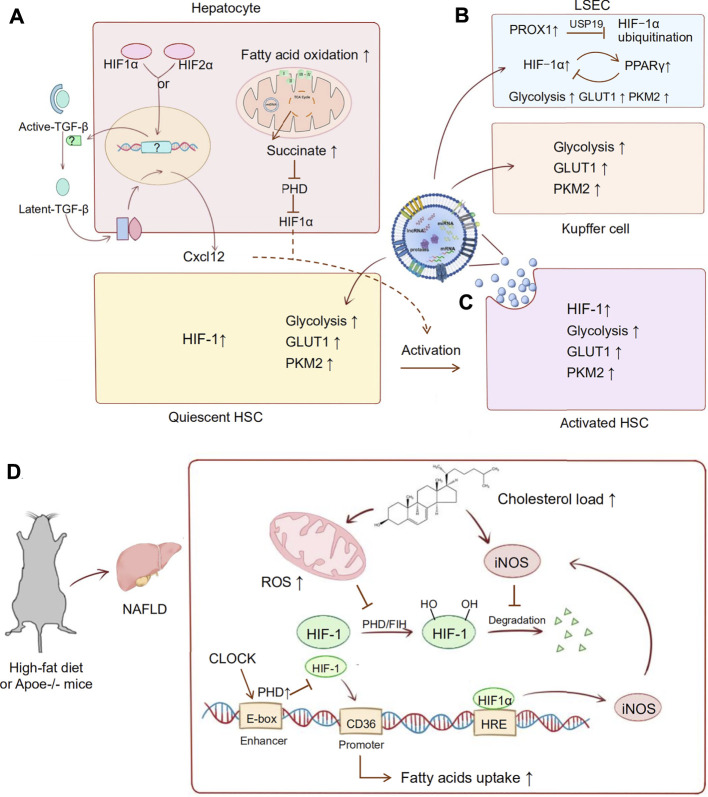
HIF pathway applied in liver fibrosis. During the development of liver fibrosis, oxygen mediated metabolic reprogramming occurs in a variety of cells. Abnormal lipid metabolism and activation of HIF in hepatocytes mediate the fibrotic pathway and the activation of HSC cells. Besides, activated HSCs occur in intracellular metabolic reprogramming, and they also mediate the metabolic transformation of other parenchymal cells. **(A)** In hepatocytes, activated HIF-1α or HIF-2α stimulates upregulation of Cxcl12 through converting latent TGF-β to active TGF-β. Succinate accumulates in hepatocytes due to enhanced fatty acid oxidation, which stabilized and activated HIF-1α through impairing PHDs. Expression of Cxcl12 and HIF-1α is involved in activation of HSCs. **(B)** In LSECs, PROX1 inhibites HIF‐1α ubiquitination *via* a deubiquitinase called USP19. A possible loop shows that HIF-1α induces PPARγ expression as a hypoxic response, then overexpressed PPARγ will inhibit HIF-1α transcription hypoxia as a negative feedback. **(C)** In activated HSCs, HIF-1 upregulates the GLUT1 and PKM2 expression in exosomes. Then these exosomes are absorbed by KCs, LSECs, and quiescent HSCs, which enhanced glycolysis of these nonparenchymal cells. **(D)** High-fat diet and Apoe knockout are common modeling methods of NAFLD. HIF-1 is also involved in metabolic disorder in the development of NAFLD to liver fibrosis. In this process, cholesterol load increased mitochondrial dysfunction and iNOS levels, which promoted HIF-1 stabilization and transcriptional activity. Then, the abnormal activation of HIF-1 promoted the production of iNOS and formed a malignant loop for fibrosis. Furthermore, HIF-1 is also involved in the circadian locomotor-related metabolic disorders in NAFLD. In CLOCK deficiency, HIF1α binds to the Cd36 promoter, promoting CD36 expression and uptake of fatty acids in the liver. High fat feeding and AP knockout mice are common modeling methods of NAFLD.

In mice fed a high-fat diet (HFD), hepatic steatosis leads to liver tissue hypoxia. The HIF1-mediated PTEN/NF-kB-p65 pathway plays a critical role in the development of NAFLD to liver fibrosis (Han et al., 2019). In an apolipoprotein E-deficient (Apoe−/−) mouse model, the circadian locomotor output cycle kaput (CLOCK) protein indirectly regulates HIF1α expression by modulating PHD protein levels. In CLOCK deficiency, HIF1α binds to the Cd36 promoter, promoting CD36 expression and uptake of fatty acids in the liver. This regulatory link among hypoxia, metabolism, and circadian locomotor promotes cirrhosis in NAFLD (Pan et al., 2020). Studies of high cholesterol diet (HCD)-induced liver fibrosis revealed that inducible nitric oxide synthase (iNOS)-mediated enhancement of fibrosis was associated with HIF1α stabilization (Anavi et al., 2015). It has been suggested that cholesterol-mediated activation of HIF-1 is ROS- and nitric oxide (NO)-dependent. Cholesterol load increased mitochondrial dysfunction and NO levels, which promoted HIF-1 stabilization and transcriptional activity (Anavi et al., 2014). Succinate, an intermediate of the tricarboxylic acid cycle, accumulates in hepatocytes due to enhanced fatty acid oxidation in fibrosis. Accumulated succinate stabilizes and activates HIF-1α by impairing PHDs, which induces inflammation and HSC activation (Cho, 2018; She et al., 2018). We summarized the details of the mechanism above in Figure 1.

This evidence indicates that the oxygen balance in liver fibrosis is disrupted, which mediates metabolic disorders and the pathological accumulation of metabolic substances. There is no doubt that HIF-mediated oxygen balance control is a potential target for metabolic liver disease.

### HIF Mediated-Immune Regulation in Liver Fibrosis

Although HIF-α is often activated in liver diseases, the roles of HIF-1 mediated-immune regulation in different liver injuries are different. In cholestatic liver disease, nuclear HIF-1α protein was present in hepatocytes, liver macrophages, and liver fibroblasts of patients with primary biliary cirrhosis and primary sclerosing cholangitis (Copple et al., 2012). A study of cholestatic mice indicated that chronic liver injury activated HIF-1α in macrophages. Activated HIF in macrophages may regulate the expression of platelet-derived growth factor-B (PDGF-B) to promote fibrosis, which induces HSC proliferation, chemotaxis, and collagen production.

In NASH mice, the significant upregulation of HIF‐1α in hepatocytes increased proportion of M2 macrophages and promoted liver fibrosis and HCC (Ambade et al., 2016). Furthermore, HIF‐1α is not only upregulated in hepatocytes, where it induces steatosis, but also in macrophages of NASH patients (Wang et al., 2019). The role of HIF-1α in macrophages in NASH was explored in the methionine-choline-deficient (MCD) diet-fed mice. Mice with stabilized HIF-1α levels in macrophages showed higher steatosis and liver inflammation. HIF-1α impaired autophagic flux in macrophages and upregulated NF-κB activation and monocyte chemoattractant protein-1 (MCP-1) production, leading to MCD diet-induced NASH (Wang et al., 2019). At the same time, digoxin was reported to be protective in inflammasome activity in macrophages and hepatic oxidative stress response in hepatocytes in NASH (Zhao et al., 2019). The protective effect was related to the downregulation of HIF-1α transactivation (Ouyang et al., 2018).

A recent article has reported the role of HIF-1α in viral hepatitis type B that HIF-1α down-regulated apolipoprotein B mRNA editing enzyme catalytic subunit 3B (APOBEC3B) and thus impaired the anti-HBV effect of A3B (Riedl et al., 2021). However, HIF-1α also induced deoxyribonucleases (DNases), which limited the duplication of hepadnaviruses (Hallez et al., 2019).

In contrast, the role of HIF-1α in chemicals-induced acute liver injury is different from its role in chronic liver diseases. Mochizuki et al. proposed a model in which liver necrotic cells could activate HIF-1α in HSCs through regional hypoxia or other mechanisms yet to be determined. HIF-1α then stimulated recruited macrophages to remove necrotic hepatocytes. In HSC-specific HIF-1α knockout, the levels of M1 macrophage activation markers and the percentage of Gr1^hi^ macrophages in the liver were reduced, which impaired the clearance of necrotic cells and promoted fibrosis (Mochizuki et al., 2014). In APAP-induced liver injury, T-cell-specific Hif-1α gene knockout mice sustained severe liver damage, which was related to the aggravated inflammatory responses by enhancing aberrant innate-like γδ T-cell recruitment and excessive neutrophil infiltration (Suzuki et al., 2018).

**FIGURE 2:**
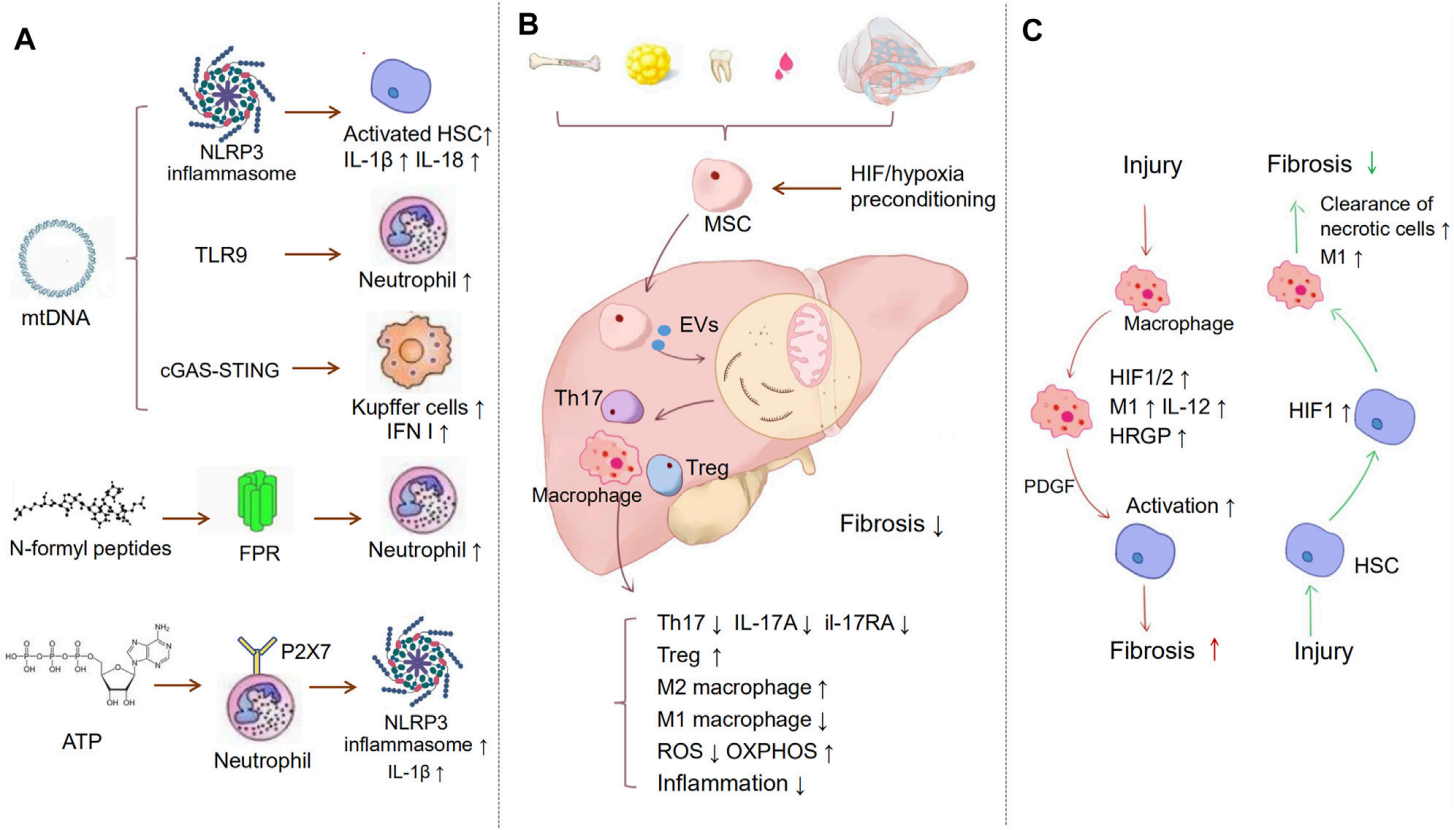
Immune regulation in liver fibrosis. **(A)** Inflammation is a major process in the development of fibrosis. MtDAMPs, including mtDNA, N-formyl peptides, and ATP, are released to extracellular space in the case of ROS-driven mitochondrial membrane permeability transition. MtDNA can activate NLRP3 inflammasomes, TLR9, and cGAS-STING, which separately induce HSCs, neutrophil, and KCs activation. NFPs are released from dysfunctional mitochondrial, binding to FPRs, which leads to the migration of neutrophil cells to the injury tissue. Released ATP binds to P2X7 receptors on neutrophils, inducing NLRP3-ASC-caspase-1 inflammasome and IL-1β secretion. **(B)** MSCs act as a bridge to prevent inflammation and oxidative stress *via* extracellular vesicles, etc. MSCs suppress the proliferation of Th17 cells and decrease the expression of IL-17A and il-17RA. Meanwhile, MSCs promote the activation of M2 macrophages and inhibit M1 macrophages activation, hypoxia-preconditioning, and HIF-1 overexpression can improve MSC therapeutic. **(C)** HIF may play different roles in different cells. The chronic liver injury activates HIF-1α in macrophages, which may regulate the expression of PDGF-B to induce HSCs activation and fibrosis. However, liver necrotic cells can also activate HIF-1α in HSCs. HIF-1α then stimulated recruited macrophages to remove necrotic hepatocytes and alleviate fibrosis.

It has also been reported that hepatocyte-specific deletion of HIF‐2α improved NAFLD‐associated fibrosis through downregulated histidine‐rich glycoprotein (HRGP). The fraction of inflammatory Ly6C^high^ hepatic macrophages, its production of IL‐12, and the expression of M1 cytokines/chemokines were significantly decreased in HIF‐2α^–/–^ mice. These findings indicated that HIF‐2α/HRGP in parenchymal cells could promote proinflammatory responses of hepatic macrophages (Bartneck et al., 2016; Morello et al., 2018). These studies suggest that although liver injury is usually accompanied by the activation of HIF, different activated cells may have opposite effects on fibrosis. We summarized the oxygen imbalance mediated-immune alterations above in Figures 2A,C.

## Therapeutic Significance of Oxygen Homeostasis in Liver Fibrosis

### Therapies Targeting Mitochondrial Dysfunction to Alleviate Fibrosis

Since increasing evidence has proven the crucial role of mitochondria in liver fibrosis, several efforts have been made to assess the efficacy of pharmacologic therapies targeting mitochondria.

Attenuated mitochondrial dysfunction, increased mitochondrial fission, decreased HSC migration and activation, and decreased oxidative stress are involved in the protective role of augmenting liver regeneration (ALR) in liver fibrosis (Song et al., 2011; Ai et al., 2018). Ming Song et al. first reported the therapeutic effect of ALR gene therapy (Song et al., 2011). The underlying mechanisms were attenuating mitochondrial dysfunction and oxidative stress and inhibiting the activation of HSCs. The results of Ai et al. (2018) were consistent with the former results. The inhibition of ALR expression aggravated liver fibrosis in mice that were administered CCl4 by promoting mitochondrial fusion and HSC migration. The inhibition of ALR may lead to increased mitochondrial Ca^2+^ influx in HSCs, resulting in HSC migration. ALR transfection inhibited F-actin assembly, retarded HSC migration, and promoted mitochondrial fission (Ai et al., 2018).

Poly (ADP-ribose) polymerase (PARP) activation was found in patients with hepatic cirrhosis, and the inhibition of PARP had antifibrotic effects (Mukhopadhyay et al., 2017). PARP inhibition or genetic deletion of PARP1 was reported to attenuate alcohol-induced hepatic oxidative stress and mitochondrial dysfunction by improving the activity of complexes I and IV (Mukhopadhyay et al., 2017). Xing Lin et al. reported that didymin could alleviate liver injury and fibrosis induced by CCl4 by inhibiting HSC proliferation and inducing apoptosis (Lin et al., 2016).

HSC apoptosis was partly mediated by MPTP opening. Didymin treatment led to cytochrome c release into the cytosol and decreased Bcl-2 expression, resulting in HSC apoptosis (Lin et al., 2016). Similarly, the curative effect of dihydroartemisinin (DHA) on liver fibrosis was also partly mediated by HSC apoptosis by releasing cytochrome c and activating the caspase pathway (Chen et al., 2016a).

Oxidative stress is a main stimulative factor of liver fibrosis, and it is a promising target. Melatonin may improve hepatic mitochondrial functions and thus reduce oxidative stress in some diseases (Coto-Montes et al., 2012; Jimenéz-Aranda et al., 2014; Agil et al., 2015). In CCl4-induced liver fibrosis rats, melatonin protected against liver fibrosis by attenuating mitochondrial dysfunction, which was manifested by improved mitophagy and mitochondrial biogenesis (Kang et al., 2016). Melatonin also attenuated lipid-mediated mitochondrial dysfunction and ROS generation in hepatocytes and improved mitochondrial respiratory functions, leading to decreased oxidative stress and inflammation and thus inhibition of HSC activation (Das et al., 2017). Another mitochondria-targeted antioxidant, mitoquinone, could attenuate liver fibrosis by reducing hepatic oxidative stress, preventing apoptosis, and promoting the removal of dysfunctional mitochondria (Turkseven et al., 2020).

It has been reported that adiponectin and its receptors enhanced fatty acid oxidation and glucose uptake and prevented the activation of HSCs induced by CCl4, thus alleviating NASH and fibrosis in mouse models (Xu et al., 2020).

Mitophagy is a selective form of autophagy that eliminates dysfunctional mitochondria (Williams et al., 2015a). It protects the liver from both acute and chronic ethanol consumption (Ma et al., 2020). Targeting mitophagy may protect the liver from acetaminophen and alcohol injury (Williams et al., 2015b). Interestingly, chronic deletion (KO) of Parkin alleviated APAP-induced liver injury, but acute knockdown of Parkin exacerbated injury (Williams et al., 2015a). This result suggested other protective pathways in the liver.

### Therapies Targeting the HIF Pathway to Alleviate Fibrosis

Since hypoxia and HIFs are considered to be important drivers of liver fibrosis, targeting HIF may be an effective treatment for fibrosis (Strowitzki et al., 2018). In mice fed with HFD, curcumin can inhibit succinate-induced HSC activation by blocking the HIF-1α signalling pathway in mouse primary HSCs (She et al., 2018). In acute liver injury Tamoxifen, an agonist of the G protein-coupled oestrogen receptor (GPER), has been confirmed to inhibit the HIF1α pathway and prevent HSC activation by a mechanical mechanism (Cortes et al., 2019). In a rat model of CCl4-induced liver fibrosis, ligustrazine alleviated hepatic injury, angiogenesis, and vascular remodelling by decreasing the level of HIF-1α (Zhang et al., 2018). The combination of celecoxib and octreotide decreased thioacetamide-induced liver fibrosis in rats by inhibiting the phosphorylation of the extracellular signal-regulated kinase (p-ERK)/HIF-1α/vascular endothelial growth factor (VEGF) pathway (Gao et al., 2016). MicroRNA-122 can protect the liver from ethanol-induced injury and fibrosis by inhibiting HIF-1α expression (Satishchandran et al., 2018). In cholestatic liver fibrosis, it has also been reported that EW-7197, a TGF-β Type I receptor kinase inhibitor, can inhibit HIF1α-induced epithelial mesenchymal transition to alleviate cholestatic liver fibrosis (Kim et al., 2016). In NASH, isochlorogenic acid B was reported to have anti-fibrosis effects by inhibiting HSC activation, attenuating oxidative stress via Nrf2, and suppressing multiple profibrogenic factors through miR-122/HIF-1α signalling pathway (Liu et al., 2019). The protective role of digoxin in steatohepatitis was related to the inhibition of PKM2/HIF-1α signalling pathway (Ouyang et al., 2018; Zhao et al., 2019).

### MSCs Act as a Bridge to Link Immunometabolism, Oxygen Homeostasis, and Fibrosis

Mesenchymal stem cells (MSCs) are pluripotent stem cells that can be induced to differentiate into several tissue cells (Chen et al., 2016b). MSC sources are diverse, such as in bone marrow, adipose tissue, placenta, amniotic tissue, cord, lung, liver, and skin (Zhuang et al., 2019). Existing studies have shown that MSC therapy is prominently effective in hepatic fibrotic diseases indirectly by regulating the immune metabolism microenvironment (El Agha et al., 2017). The secretion of IL-17A from Th17 cells can promote fibrosis by activating fibroblasts (Huang et al., 2019; Hu et al., 2020). BM-MSCs inhibited liver fibrosis by decreasing the expression of IL-17A and IL-17RA and the serum levels of IL-17 in the liver (Farouk et al., 2018). Milosavljevic et al. also confirmed that MSC-conditioned medium (MSC-CM) promoted the expansion of CD4^+^-FoxP3^+^-IL-10^+^-T regulatory cells and suppressed the proliferation of Th17 cells, which attenuated liver fibrosis (Dong et al., 2020).

Furthermore, BM-MSC transplantation promoted the activation of M2 macrophages expressing matrix metalloproteinase 13 (MMP13) and inhibited M1 macrophage activation. Meanwhile, MSCs reduced the expression of proinflammatory cytokines and increased the expression of anti-inflammatory cytokines (van der Helm et al., 2018; Luo et al., 2019; Yu et al., 2019). Increasing mitophagy and reducing mitochondrial ROS to restrict the inflammatory activation of macrophages may be critical mechanisms by which MSCs inhibit inflammation (Li et al., 2018). In response to oxidative stress, MSCs can transport depolarized mitochondria to macrophages through extracellular vesicles (Phinney et al., 2015). Mitochondrial transfer also promotes an anti-inflammatory macrophage phenotype by enhancing oxidative phosphorylation (Morrison et al., 2017). Existing studies also confirmed that hypoxia preconditioning and HIF-1 overexpression significantly improved MSC therapy (Martinez et al., 2017). MSCs cultured under hypoxic conditions presented an enhanced therapeutic effect on liver cirrhosis, which promoted macrophage polarity to an anti-inflammatory phenotype via prostaglandin E2 (PGE2) expression (Kojima et al., 2019). In summary, MSC treatment is emerging as a connecting bridge to drive immune and metabolic regulation and oxygen balance in the fibrotic microenvironment. We summarized the details of mechanism above in Figure 2B.

## Conclusion and Future Perspectives

**FIGURE 3:**
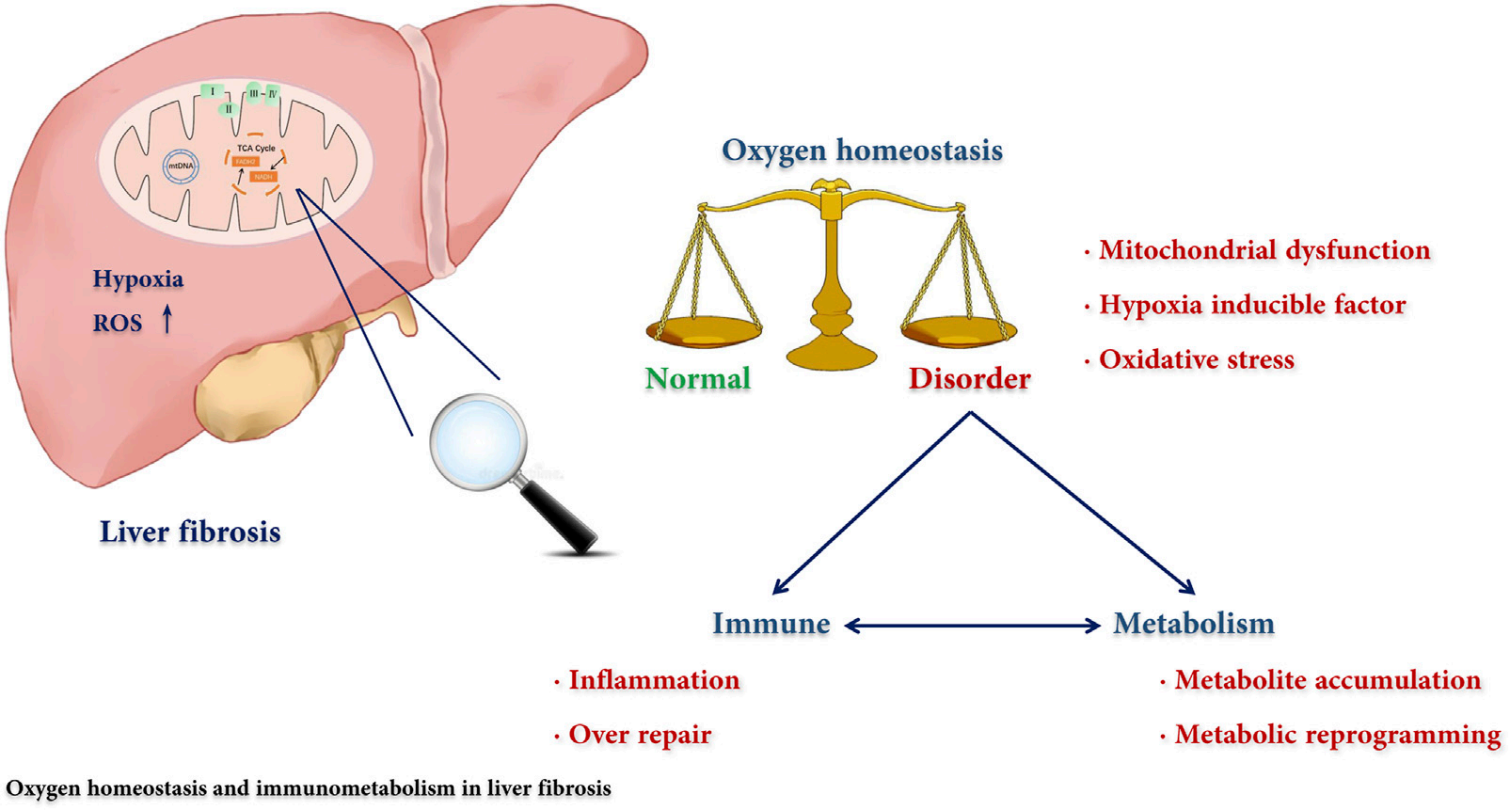
The regulatory mechanism for immunometabolism and oxygen homeostasis in liver fibrosis. The disorders of immunity and metabolism are cross-linked in liver fibrosis and play a central role in the pathogenesis. We reviewed the effect of the destruction of oxygen homeostasis on liver fibrosis and described how oxygen participated in this process through affecting the immune-metabolism axis.

Mitochondrial dysfunction, hypoxia, inflammation, and metabolic reprogramming are widespread in fibrotic diseases. Here, we have reviewed the regulatory mechanism for immunometabolism and oxygen homeostasis in liver fibrosis (Figure 3) as well as potential novel targets for antifibrotic therapies. A special metabolic immune microenvironment mediated by oxygen is described, which deeply affects the balance of tissue damage and repair. The process of fibrosis is closely related to metabolic and immune disorders, which are usually induced by the destruction of oxygen homeostasis, including mitochondrial dysfunction, oxidative stress, and hypoxia signalling pathway activation. On the one hand, destruction of oxygen homeostasis promotes oxidative stress and releases inflammatory mediators, forming a loop with an inflammatory response and cell damage. On the other hand, cell metabolic reprogramming affects the activation of immune cells and fibroblasts, epithelial mesenchymal transformation, and angiogenesis and further promotes the development of fibrosis. Furthermore, we noticed that hypoxia-induced metabolic reprogramming of immune cells and other fibrosis-related cells is an emerging research direction, but there is still a gap to be filled in the liver fibrosis field. In summary, as immunometabolism and oxygen homeostasis are relatively new research directions, the mechanism, function, and potential clinical application in liver fibrosis need and deserve further investigation.

## Glossary

ECM: Extracellular matrix
HSCs: hepatic stellate cells
DAMPs: danger-associated molecular patterns
HIF-1α: hypoxia inducible factor-1α
ATP: adenosine-triphosphate
ROS: reactive oxygen species
mtDNA: mitochondrial DNA
NAFLD: non-alcoholic fatty liver disease
PPAR γ: peroxisome proliferator ‐activated receptor ‐gamma
PGC ‐1α: peroxisome proliferator ‐activated receptor ‐gamma coactivator ‐1alpha
NRF: nuclear respiratory factor
TFAM: mitochondrial transcription factor A
CCl4: carbon tetrachloride
PSO: pomegranate seed oil
EET-A: epoxyeicosatrienoic acid-agonist
Mfn1: fusion proteins mitofusin 1
OPA1: optic atrophy 1
Drp1: dynamin-related protein 1
PM2.5: Particulate matter ≤ 2.5 μm
HKDC1: HK domain-containing 1
LA: lipoic acid
mtDepo: mitochondrial depolarization
BNIP3: Bcl2/adenovirus E1B 19 kDa protein-interacting protein 3
mtDAMPs: mitochondrial DAMPs
SOD: superoxide dismutase
CAT: catalase
GSH: glutathione
NLRP3: NACHT, LRR and PYD domains-containing protein 3
MAPK: mitogen-activated protein kinase
PHD: prolyl hydroxylase domain
VHL: von Hippel‐Lindau
HRE: hypoxia response element
ROCK1: Rho-associated coiled-coil-forming kinase 1
Cxcl12: chemokine ligand 12
TGF-β: transforming growth factor β
PROX1: prospero homeobox protein 1
USP19: ubiquitin specific peptidase 19
LSECs: liver sinusoidal endothelial cells
PPARγ: peroxisomal proliferator receptor γ
GLUT1: glucose transporters 1
PKM2: pyruvate kinase isozymes M2
NPCs: non-parenchymal cells
HFD: high-fat diet
Apoe-/-: apolipoprotein E-deficient
CLOCK: circadian locomotor output cycles kaput
HCD: high cholesterol diet
iNOS: inducible nitric oxide synthase
NO: nitric oxide
SI: sterile inflammation
HMGB-1: high mobility group box-1
KC: Kupffer cell
RAGE: receptor for advanced glycation end-products
HCC: hepatocellular carcinoma
PRRs: pattern recognition receptors
APCs: antigen-presenting cells
TLR9: Toll-like receptor 9
NFPs: N-formyl peptides
NASH: nonalcoholic steatohepatitis
IL: interleukin
PA: palmitic acid
APAP: acetaminophen
miR-223: microRNA-223
cGAS: cyclic GMP-AMP synthase
STING: stimulator of interferon genes
NF-κB: nuclear factor-κB
IFN I: type I interferon
FPRs: formyl peptides receptors
SIRS: systemic inflammatory response syndrome
IRI: ischemia / reperfusion injury
PDGF-B: platelet-derived growth factor-B
MCD: methionine-choline-deficient
MCP-1: monocyte chemoattractant protein-1
APOBEC3B: apolipoprotein B mRNA editing enzyme catalytic subunit 3B
DNases: deoxyribonucleases
HRGP: histidine‐rich glycoprotein
ALR: augmenter of liver regeneration
DHA: dihydroartemisinin
GPER: G protein-coupled estrogen receptor
p-ERK: phosphorylation of extracellular signal-regulated kinase
VEGF: vascular endothelial growth factor
MSCs: mesenchymal stem cells
MSC-CM: MSC-conditioned medium
MMP13: matrix metalloproteinase 13
PGE2: prostaglandin E2
